# Upcycling Chili Pepper Processing Waste into a Capsaicinoid-Rich Spicy Oleoresin Food Ingredient: A Review of Ultrasound-Assisted Extraction Studies

**DOI:** 10.3390/foods15213789

**Published:** 2026-10-22

**Authors:** António Toscano, Patrícia Fradinho, Filipa V. M. Silva

**Affiliations:** 1 LEAF—Linking Landscape, Environment, Agriculture and Food Research Center, Instituto Superior de Agronomia, Universidade de Lisboa, Tapada da Ajuda, 1349-017 Lisboa, Portugal; antonio.graca.4@gmail.com; 2 Colab4Food—Collaborative Laboratory for Innovation in the Agri-Food Sector, 4485-655 Vairão, Vila do Conde, Portugal; 3 Associate Laboratory TERRA, Instituto Superior de Agronomia, Universidade de Lisboa, Tapada da Ajuda, 1349-017 Lisboa, Portugal

**Keywords:** *Capsicum*, pungent, spice, piquant, capsaicin, by-product, sonication, sustainability, circular bioeconomy

## Abstract

The growing demand for spicy foods and industrial production of hot sauces and spicy condiments from chili pepper (*Capsicum* spp.) generates substantial quantities of by-products, mainly composed of placenta, seeds, and fibrous tissues, which retain significant concentrations of capsaicinoids and other valuable phytochemicals. These residues represent an underexploited resource for the development of high-value food ingredients. This review critically examines the valorization of chili pepper processing by-products through the recovery of capsaicinoid-rich oleoresins, with particular emphasis on ultrasound-assisted extraction (UAE) using ethanol-based solvents. The ultrasound-enhanced mass transfer and processing parameters, extraction yields, oleoresin spiciness, and scalability challenges were discussed. Past studies showed UAE substantially reduces extraction times while improving process efficiency and enabling the production of oleoresins with high pungency. Sonication times of 8–20 min and temperatures between 35 and 65 °C produced oleoresin yields ranging from 19 to 28% (dry basis) depending on the chili pepper variety and processing conditions. Pungency values reached 953,920 Scoville Heat Units for oleoresin obtained from the by-product of Carolina Reaper pepper variety. Limitations of ultrasound technology regarding process standardization and scale-up were also discussed. Overall, UAE emerges as a promising technology for transforming chili pepper industrial waste into natural spicy ingredients.

## 1. Introduction to Spicy Foods, Spicy Sauces and Spicy Ingredients Obtained from Chili Peppers

The global market for spicy foods has experienced sustained and accelerated growth, largely driven by increasing consumer demand for natural products, diverse flavor profiles, and novel sensory experiences linked to exotic gastronomy [1]. In 2025, the global spicy food market alone was valued at approximately USD 5.7 billion [2], and it is projected to reach USD 8.4 billion by 2034, representing a compound annual growth rate of around 4.27%. Although widely used, the term “spicy food” lacks a standardized definition. Broadly, it may refer to foods seasoned with aromatic spices such as cumin, black pepper, ginger, or garlic, as well as ingredients responsible for pungency, particularly chili peppers and their derivatives [3].

Chili peppers or hot peppers (*Capsicum* spp.), are the fruits of a native plant from South and Central America, belonging to the Solanaceae family. This is one of the most cultivated horticultural crops worldwide since the age of discovery [4]. In the 16th century, Portuguese traders first introduced this plant in Africa and Arabia, and then in Asia (India, Malaysia, Sri Lanka) through their trading posts in Goa and Malacca. They are distinguished by their vibrant red-to-orange colors, diverse morphologies, and characteristic pungent and intense flavor [5,6]. Main cultivated chili pepper species are *C. annuum*, *C. chinense* (e.g., cultivars Red Habanero and Carolina Reaper), *C. baccatum*, *C. frutescens*, and *C. pubescens*, although *C. annuum* includes pungent varieties such as Jalapeño and non-spicy sweet bell peppers. Chili peppers have acquired strong cultural and gastronomic significance, exemplified by the recognition of traditional Mexican cuisine, an Intangible Cultural Heritage of Humanity by UNESCO in 2010, where chili peppers are cornerstone ingredient [7]. Foods containing chili peppers are especially influential in driving market growth, as they are often associated with positive sensory experiences and positive emotional responses such as stress relief and stimulation, which frames current consumer trends [8]. In this context, the food industry has increasingly utilized chili peppers as key commodities in the development of convenient, flavor-rich, and concentrated spicy products. Hot sauces and spicy condiments represent the most dominant and widely consumed product category [7]. The global sector is mainly led by major producing countries such as the United States, China, India, and Japan [9], collectively producing approximately 8 million units of spicy sauces annually. Leading brands such as Tabasco (McIlhenny), Frank’s RedHot (McCormick), and Sriracha (Huy Fong) occupy significant market shares [10].

**Figure 1:**
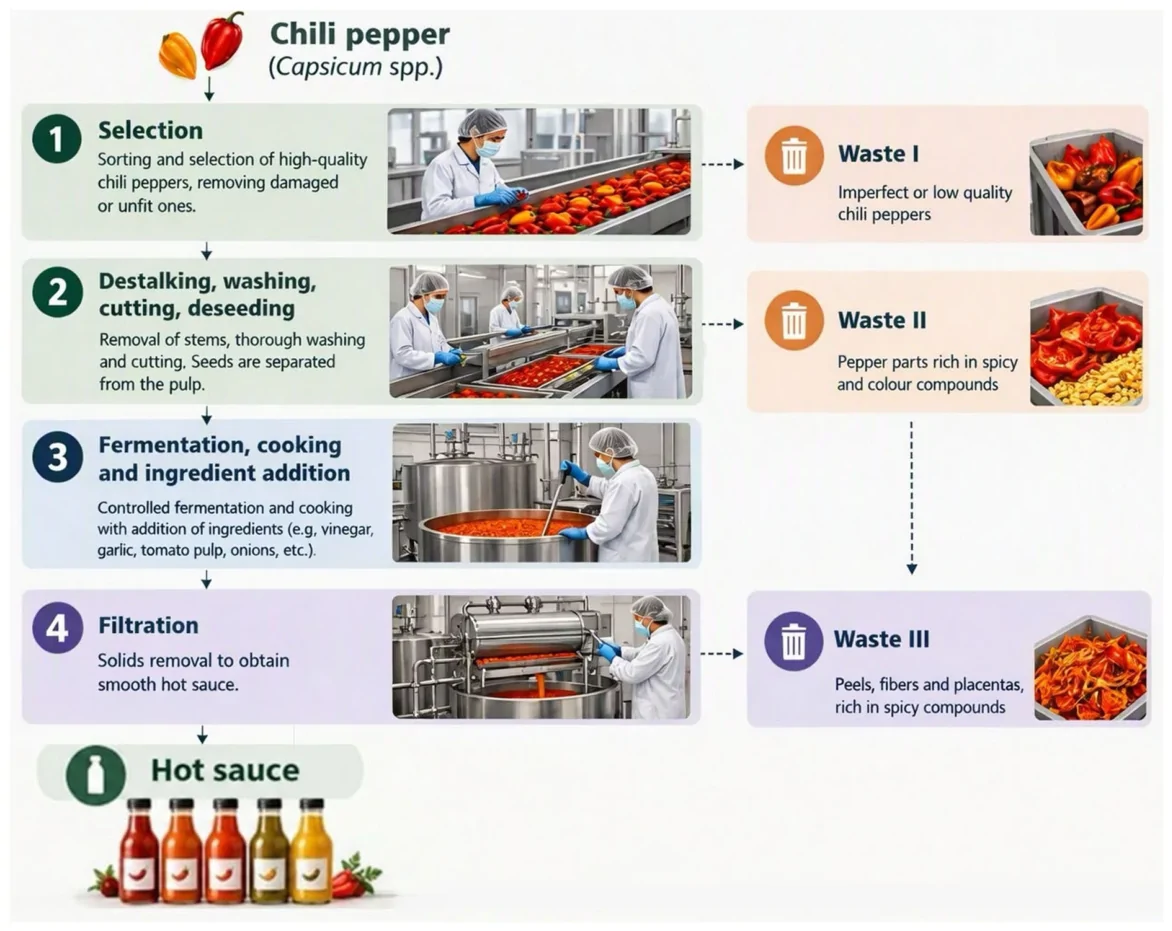
Process diagram for the industrial production of spicy sauce from chili peppers, highlighting the generation of waste streams for each production step (This illustration was generated by the authors and improved using Microsoft Copilot Version bizchat.20261019.61.1).

Spicy food sauces and ingredients are typically manufactured using pungent oleoresins and pulps obtained from *Capsicum* species such as *Capsicum annuum*, *Capsicum frutescens*, and *Capsicum chinense* [11]. Processing commonly involves blending chili peppers with complementary ingredients (e.g., garlic, onion, tomato derivatives, vinegar, herbs, and salt), followed by thermal processing, filtration, or prolonged lactic acid fermentation, which may extend the shelf-life from several months to years (Figure 1). As the primary raw material for hot sauce production, fresh chili peppers quality attributes such as pulp content, ripeness, color, pungency, and the absence of blemishes or insect damage, is very important [12].

Beyond their spicy properties, chili peppers can also provide natural food colorants. The pigments responsible for red, orange and green colors are capsanthin, capsorubin (*Capsicum* exclusive carotenoid pigments), beta carotene and some chlorophylls, respectively [13]. Most of the pigments are extremely relevant as their applications are extended from food dye (red-to-orange tones) to functional effects (e.g., protecting food from oxidative stress), while enhancing nutritional value (these carotenoids are retinol “Vitamin A” precursors), altogether leveraging overall market value and consumer interest [14].

**Figure 2:**
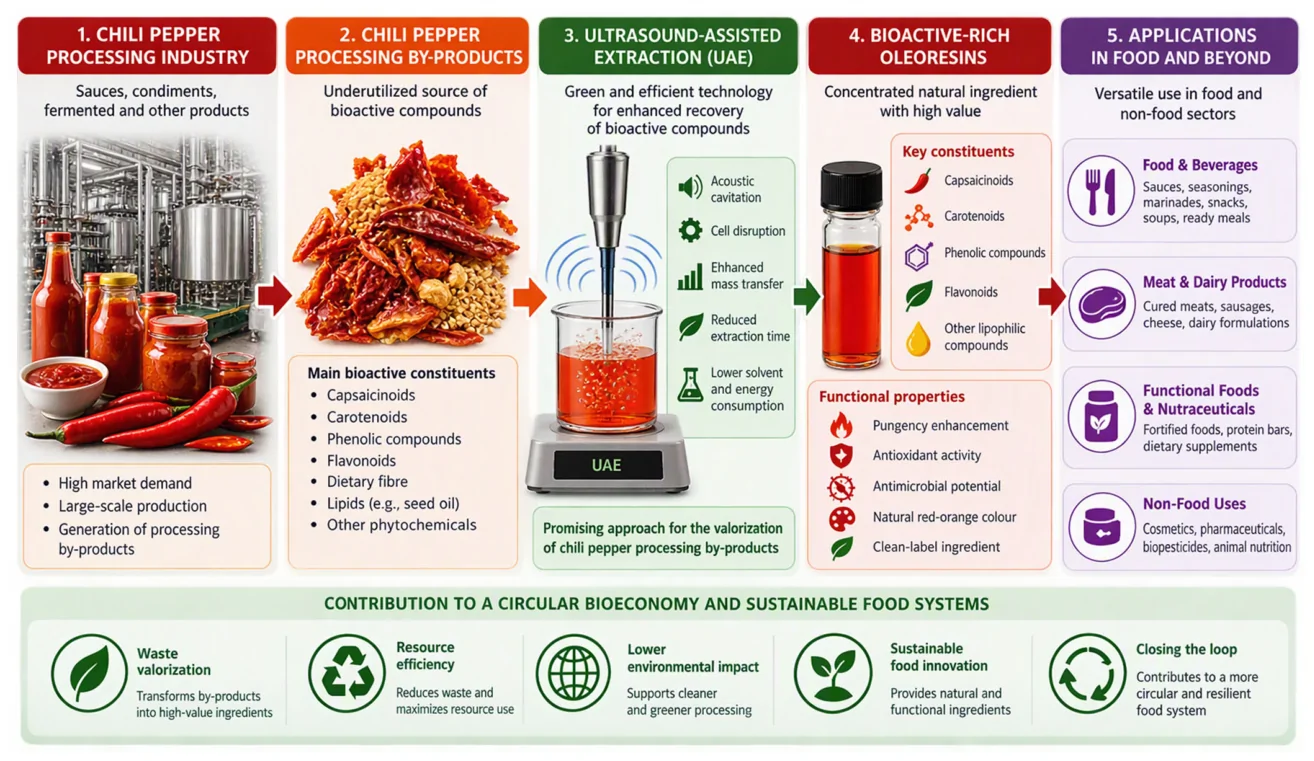
Valorisation of chili pepper processing by-products through ultrasound-assisted extraction (UAE), highlighting the conversion of capsaicinoid-rich residues into food ingredients within a circular bioeconomy approach (This conceptual illustration was generated using Microsoft Copilot Version bizchat.20261019.61.1, based on prompt instructions and information provided by the authors).

The growing market demand for natural spicy ingredients has raised interest in alternative sources of spicy oleoresins rich in capsaicinoids. Chili pepper oleoresin is a concentrated extract obtained from spicy *Capsicum* fruits, containing the characteristic lipophilic compounds responsible for their pungent flavor and color. Spicy oleoresins are particularly rich in capsaicinoids, mainly capsaicin and dihydrocapsaicin, which provide the characteristic heat. In this context, by-products generated during chili pepper processing have emerged as attractive feedstocks for the sustainable production of high-value oleoresins for food and non-food applications, contributing to waste reduction and circular bioeconomy strategies (Figure 2). Figure 2 provides an overview of the valorization pathway discussed throughout this review. Although reviews focusing on chili pepper oleoresins, extraction and quantification of capsaicinoids, and benefits, are available in the literature, a review oriented exclusively to ultrasound-assisted extraction, in particular from chili pepper processing by-products, was not carried out. Therefore, the main objective of this review was to evaluate the application of ultrasound technology to produce oleoresins from chili pepper by-products for their subsequent use as food ingredient by the food industry. In addition, section two covers capsaicinoids (the main class of compounds responsible for pungency attribute), capsaicinoids/oleoresin biological activities and potential food, agriculture and pharmaceutical applications.

## 2. Spicy Capsaicinoid Compounds: Biological Properties and Applications

### 2.1. Capsaicin and Dihydrocapsaicin, and Assessment of Spiciness Intensity

**Figure 3:**
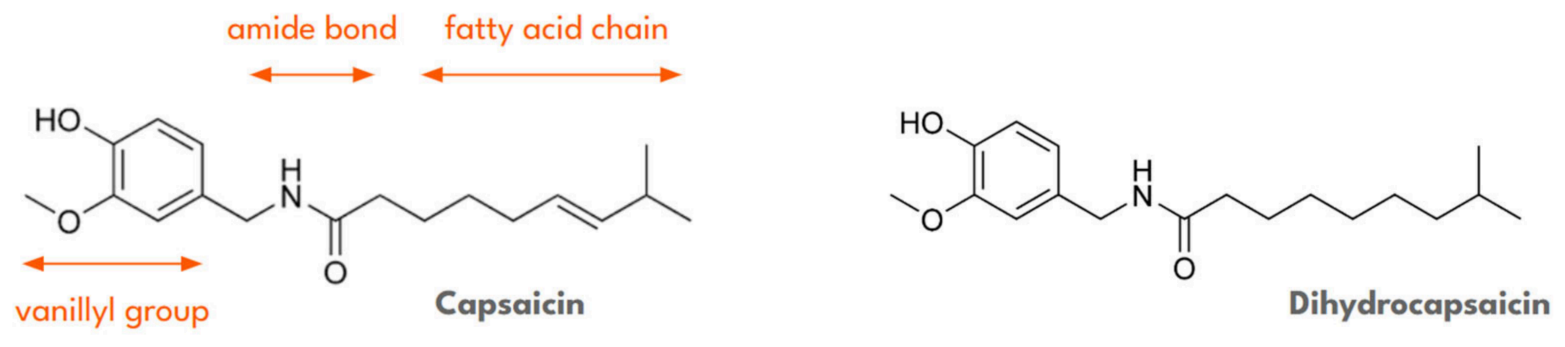
Capsaicin and dihydrocapsaicin chemical structures.

Capsaicinoids are the phytochemical compounds responsible for the appreciated spice character, and they are mostly located in the pepper fruit placenta (Figure 3). From a commercial and functional perspective, capsaicinoids represent the most valuable group of alkaloid bioactive compounds exclusive to chili peppers and their derived products. These compounds are responsible for the characteristic pungency of chili peppers, which results from the activation of pain receptors known as transient receptor potential vanilloid 1 (TRPV1) [15]. This receptor-mediated interaction produces the typical warm and burning trigeminal sensations perceived in the oral cavity and nasal mucosa, contributing to the distinctive sensory experience and hedonic responses associated with spicy food consumption [8]. Among the capsaicinoid family, capsaicin and dihydrocapsaicin (Figure 3) are the predominant compounds, together accounting for approximately 90% of the total pungent constituents in chili peppers. Other minor members of the capsaicinoids family include nordihydrocapsaicin, homocapsaicin, and homodihydrocapsaicin, which generally exhibit lower pungency levels [16,17]. Structurally, capsaicinoids share a common chemical motif consisting of an aromatic vanillyl group linked via an amide bond to a fatty acid hydrophobic chain of variable length and saturation (Figure 3).

This amphiphilic structure explains their affinity for lipid matrices and their functional behavior in aqueous food systems. Biosynthetically, capsaicinoids are formed through the condensation of phenolic precursors such as vanillyl acid with fatty acid derivatives naturally present in chili peppers, and their molecular structure is closely associated with both sensory intensity and biological activity [18]. The diversity and relative concentration of capsaicinoids largely determine the pungency of different *Capsicum* cultivars. *Capsicum annuum* L., the most cultivated pepper species, includes sweet pepper varieties and also chili pepper (e.g., Jalapeño), where generally non-pungent *Capsicum* varieties such as sweet pepper do not contain capsaicinoids. Spice levels and burning sensation depend on the chili pepper variety (or cultivar), maturity, and growth conditions. To quantify pungency, Wilbur Scoville introduced the Scoville Heat Unit (SHU) scale in 1912, which measures the degree of dilution required for capsaicinoids to become sensorially undetectable [19]. Pure capsaicin and dihydrocapsaicin correspond to a theoretical value of 16,000,000 SHU [19]. In practice, pungency varies widely among cultivars, ranging from approximately 33,000 SHU in milder varieties such as Jalapeño (*Capsicum annuum* L.) to around 860,000 SHU in Habanero (*Capsicum chinense* Jacq.), and exceeding 1,300,000 SHU in extremely pungent cultivars such as Carolina Reaper (*Capsicum chinense* Jacq., hybrid). The growing consumer demand for pungent foods has stimulated breeding programs aimed at developing cultivars with progressively higher capsaicinoid contents [11,20], reinforcing the economic relevance of capsaicinoid-rich products. Although the Scoville scale remains widely recognized, its reliance on sensory evaluation introduces limitations in terms of precision and reproducibility [21]. Consequently, analytical techniques have been developed for a more accurate quantification of capsaicinoids, with high-performance liquid chromatography (HPLC) being one of the most used [22], followed by enzyme-linked immunosorbent assays (ELISA), and methods for direct analysis in real time [23]. Despite these advances, the SHU scale continues to be broadly used as a practical benchmark for classifying chili peppers, particularly because it allows capsaicinoid concentration data to be translated into intuitive and easily interpretable values for both researchers and industry stakeholders [24,25].

### 2.2. Biological Properties of Capsaicinoids and Applications of Capsicum Oleoresins

Table 1 summarizes reported biological properties of capsaicinoids and potential food and non-food applications of *Capsicum* oleoresins. The level of evidence varies across application domains and ranges from in vitro studies to animal and human investigations. The main application of capsaicinoids is to confer pungency to foods, food condiments and food sauces, in order to enhance this flavor attribute. The two main pathways to introduce chili peppers’ spice essence into food products is by directly incorporating pepper mass or by dissolving its extract-derived oleoresin.

*Capsicum* oleoresins are lipophilic extracts obtained from chili peppers, rich in capsaicinoids and carotenoids, and are characterized by parameters such as capsaicinoid content (related to pungency intensity, SHU) and color value [3,43]. Chili pepper capsaicinoid-rich oleoresins favor food formulations with less salt without changing the consumer perception of salty taste [29]. These molecules functional properties are, in fact, aligned with consumer trends for natural products, i.e., non-synthetic food additives corroborating the development of natural food formulations [44]. In addition to spice character, *Capsicum* oleoresins inhibit the growth of undesirable microorganisms, promoting shelf-life extension [34]. Antimicrobial activity has been reported against foodborne microorganisms, namely bacteria (*Staphylococcus aureus*, *Bacillus subtilis*, *Escherichia coli*, *Pseudomonas aeruginosa*), yeast (*Candida albicans*) and mold (*Aspergillus niger*), with mechanisms involving membrane disruption, metabolic interference, and biofilm inhibition, displaying bacteriostatic or bactericidal effects depending on concentration and food matrix [31,37,45,46].

Literature also indicates the efficacy of capsaicinoids and *Capsicum* derived extracts in improving oxidative stability of edible oils and lipid-rich matrices subjected to thermal stress, supporting their positioning as effective natural alternatives to synthetic antioxidants [28,44]. These protective effects are enhanced when capsaicinoids act synergistically with other chili-derived bioactives, such as phenolic compounds and carotenoids, enabling significant inhibition of lipid peroxidation [27,47,48].

Owing to the presence of capsaicinoids and other bioactive constituents, chili peppers have also become high-value commodities for the pharmaceutical industry (Table 1). Since antiquity, chili peppers have been used not only as flavor enhancers but also in ritualistic practices and traditional medicine, particularly for pain relief [4,49]. Upon consumption, capsaicinoids can cause physiological responses linked to metabolic regulation, through the induction of thermogenesis (rapid metabolism acceleration, inducing rising body temperature), thereby significantly increasing energy expenditure [17,41]. Capsaicin might be an anti-obesity factor, useful for developing nutritionally balanced food products while adhering to a healthier lifestyle. Beyond their metabolic action, capsaicinoids exhibit other pharmacological activities, including analgesic and anti-inflammatory effects, as well as chemopreventive potential associated with anticancer and antitumoral mechanisms [17,50]. Their ability to scavenge reactive oxygen species further contributes to cellular protection, reinforcing their role in oxidative stress mitigation and overall cellular defense systems [51,52]. Nevertheless, most studies are pre-clinical and more investigation is required for pharmacological effects. In addition, the magnitude and reproducibility of these effects remain dependent on the capsaicinoids’ concentration in the oleoresin and the application conditions.

## 3. Upcycling Waste from Chili Pepper Sauce Industry into Valuable Oleoresin Using Ultrasound-Assisted Extraction (UAE)

The previous section reviewed and discussed the importance of *Capsicum* oleoresin. In this section the ultrasound-assisted extraction (UAE) of oleoresin from chili pepper by-products and whole chili peppers using ethanol-based solvents was reviewed.

### 3.1. Perspectives on Waste Valorisation Towards Economic Circularity and Sustainability

**Figure 4:**
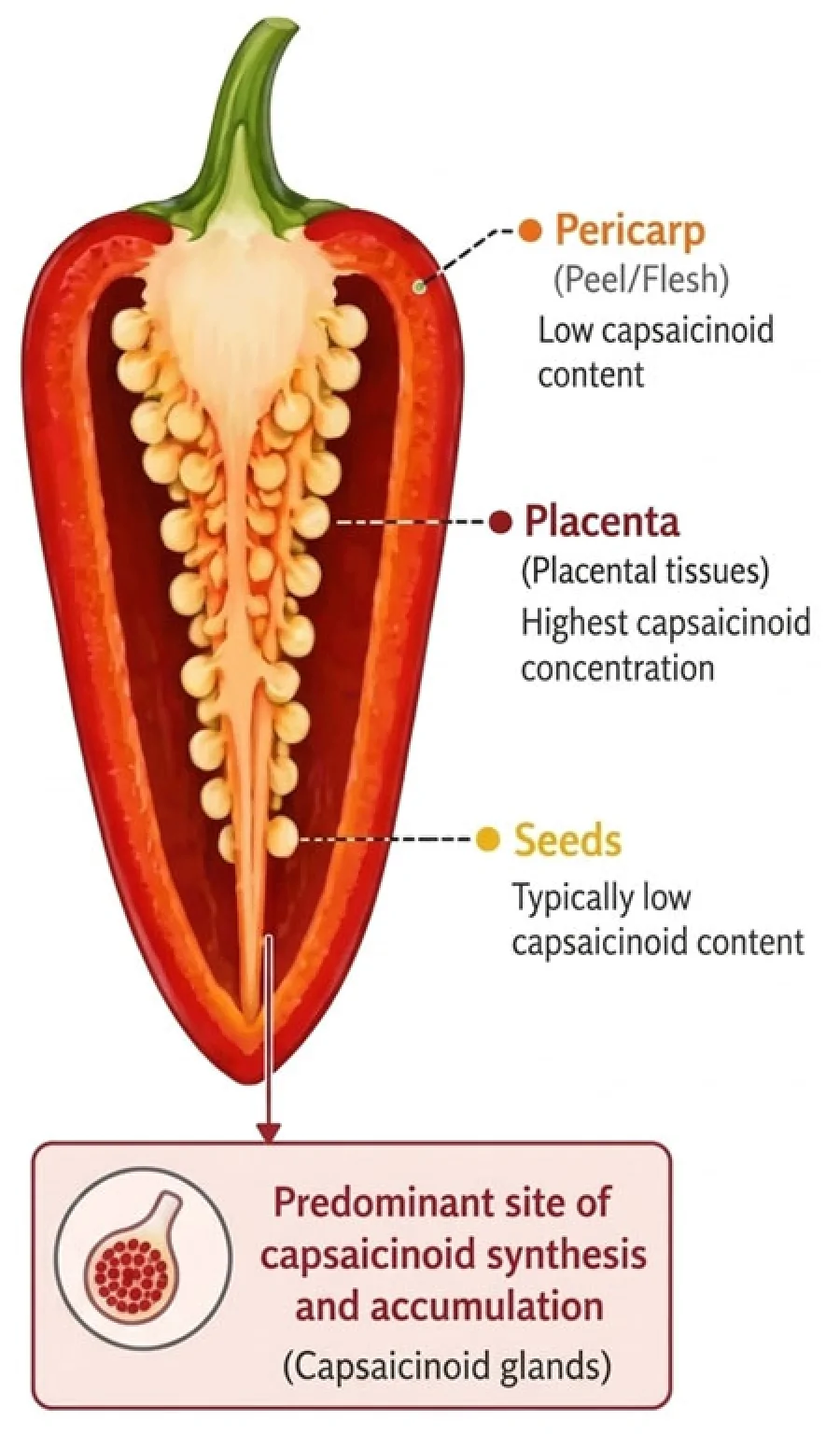
Distribution of capsaicinoids within chili pepper tissues and relative capsaicinoid concentration in different fruit locations. The relative concentration levels shown are based on information reported in the literature [56,57] and are intended as a qualitative representation (this illustration was generated using Microsoft Copilot Version bizchat.20261019.61.1, based on author-defined prompts).

Despite the high commercial value of chili peppers and their derived compounds, approximately 18% of global chili pepper production is lost or wasted [53]. This loss is largely associated with the increasingly strict quality standards required for fresh chili pepper export markets and for the industrial production of sauces and condiments [38,53]. In addition to raw material losses, the industrial processing of chili peppers generates significant amounts of waste (Figure 1). During the operations of chili pepper selection, destalking, and deseeding, followed by filtration and pulping (steps used to separate the liquid sauce from solid fractions), approximately 5–20% of the processed pepper material is converted into waste streams [43,54] (Figure 1). These residues consist largely of non-edible semi-solid fractions, such as placenta tissue, fibers, and seeds [38] (Figure 4). Chili pepper processing waste still contains capsaicinoids [53,55]. The high concentration of capsaicinoids in processing waste is mainly attributed to the presence of the placental tissues located in the fruit central axis (Figure 4), where nearly 90% of capsaicinoids are synthesized and stored [56,57]. Consequently, by-products containing placental material represent particularly attractive feedstocks for the recovery of capsaicinoid-rich oleoresins.

Animal feed is one of the potential applications. However, the European Food Safety Authority in 2025 raised concerns regarding its toxicity for animal feed production, limiting its concentration to a maximum of 6.5 mg/kg of feed for poultry, porcine species, ruminants, camelids, equines, leporids, salmonids, minor finfish, ornamental birds and fish, and dogs, whereas a maximum of 5.3 mg/kg was considered safe for cats and other species feed [58]. Despite these concerns, some food companies redirect chili processing residues to agricultural fields in order to introduce nutrients in the soil. Although composting has proven effective for residues from sweet pepper waste [59], its suitability for chili pepper organic materials is questionable due to antimicrobial properties of chili pepper derived waste, which may impact soil microbial activity and ecosystems when improperly managed [60]. While conventional linear economic models consider such waste as having limited value, circular economy approaches reinterpret these streams as by-products that can be reintegrated into the food value chain [61]. In this context, valorisation and upcycling strategies reduce disposal costs and generate additional value, particularly in large-scale processing industries [62]. Consistent with these objectives, research initiatives funded by the European Union’s Horizon 2020 program have prioritized the investigation of plant-based by-products as sources of high-value extracts, enriched fractions, and purified bioactive compounds for applications in the food and pharmaceutical sectors [26,63]. Chili pepper residues may serve as low-cost ingredients rich in bioactive compounds, fiber, vitamins, and proteins, enabling their use in the development of value-added food products such as spicy flours and condiments [64,65]. From a technological perspective, the extraction of spicy bioactive oleoresins represents one of the most promising strategies, as *Capsicum* oleoresins retain their biological value regardless of whether they originate from fresh fruits or processing waste [12,38,53]. From a strategic and ecofriendly standpoint, the on-site valorisation of chili by-products into oleoresins reinforces closed-loop supply chains, reduces transport-related emissions, and supports low-carbon production systems of concentrated extracts, especially when adopting green, novel and efficient technologies, aiming to a zero waste, zero emissions and low carbon-foot print approach [66].

The application of the chili *Capsicum* oleoresins into foods has been explored. Oleoresins derived from Jalapeño (*Capsicum annuum*) by-products were incorporated into cheese formulations, with favorable consumer acceptance (average sensory score of 7 out of 9) [67]. These results highlight the potential of chili-derived oleoresins as functional ingredients for the development of innovative food products based on industrial by-products, supporting circular and sustainable production systems within the spicy sauce/condiment industry.

The industrial application of oleoresins recovered from chili pepper processing by-products also requires consideration of food safety and regulatory aspects. Although these residues originate from food-grade raw materials, their transformation into food ingredients must ensure compliance with applicable food legislation, including requirements related to hygiene, traceability, contaminant control and solvent residues. The use of food-grade extraction solvents, standardized processing conditions and appropriate quality assurance systems is therefore essential to guarantee ingredient safety and consistency.

### 3.2. Methodology for Selecting the Original Papers Reviewed

A literature search of original results papers in Scopus was conducted from before 1960 until September 2026. Review papers were not included. This review aimed to critically evaluate the potential of ultrasound-assisted extraction (UAE) for the recovery of capsaicinoid-rich oleoresins from chili pepper sauces industrial processing by-products (e.g., from sauce and condiments production). Therefore, studies focused on the extraction of oils from leaves, seeds, stalks or other plant materials not representative of industrial chili pepper processing by-products were excluded. Sweet pepper studies were also excluded. The search of documents was carried out on article titles, abstracts and keywords based on combinations (and/or) of the following keywords: chili pepper, spicy pepper, spicy sauce, by-product, residue, chili waste, oleoresin, ultrasound extraction, capsaicin, capsaicinoid. Studies using ethanol-based solvent were considered eligible for inclusion, but not methanol. Given the limited number of publications directly addressing UAE of genuine chili pepper processing by-products, studies involving whole chili pepper fruits were also considered when relevant to support the discussion. This distinction was maintained throughout the review whenever cross-study comparison of results was carried out.

### 3.3. Ultrasound-Assisted Extraction of Capsaicinoid-Rich Capsicum Oleoresins

In the recovery of thermolabile bioactive compounds from agro-industrial by-products and other organic matrices, rapid, low-cost and sustainable technologies have gained increasing prominence over conventional extraction methods. With respect to chili pepper processing by-products, capsaicinoids generally exhibit higher thermal stability than carotenoids and certain phenolic compounds [48]. Traditional techniques often present limitations such as lower extraction efficiency, longer processing times, more solvent required, and a comparatively larger carbon footprint [43]. In response to these challenges, alternative extraction approaches including pulsed electric fields (PEF), microwave-assisted extraction (MAE), supercritical fluid extraction (SFE) and ultrasound-assisted extraction (UAE) have been attempted to enhance extraction [66]. Among these techniques, UAE stands out due to its commercial implementation, relatively low cost, scalability, rapid processing times, and operational simplicity [68]. Furthermore, the physical structure of chili pepper waste, containing different types of solid tissues (seeds, placenta, peels) also favors the use of ultrasound instead of other alternative extraction technology. Over the last two decades, research studies have further highlighted UAE as one of the most mature extraction technologies for industrial implementation, owing to its favorable balance between extraction efficiency, processing time, operational simplicity and scalability when compared with other intensification technologies [69,70,71]. Moreover, the technology requires minimal modifications during scale-up, making it particularly suitable for large scale recovery of target compounds [16,66]. When operated under optimized conditions, UAE can achieve higher extraction yields, while simultaneously reducing energy consumption by as much as 95%, representing a critical advantage in industrial processing scenarios where time and energy are key-economy factors [43,72].

Ultrasound-assisted extraction is widely used to enhance food processing operations and promote the recovery of valuable compounds from food matrices and agro-industrial by-products [68]. The technique relies on the propagation of acoustic waves of high intensity (10 to 100 W·cm^−2^) and low frequency (20–40 kHz) through liquid or semi-solid media. The ultrasound waves generate oscillatory particle motion and induce the phenomenon of acoustic cavitation, which occurs when alternating compression and rarefaction cycles, reducing the local pressure below the vapor pressure of the liquid, resulting in the formation of microscopic vapor bubbles that grow and subsequently collapse violently. This phenomenon causes plant cells’ disruption improving the extraction efficiency in comparison to conventional solvent extraction methods. The efficiency of sonication is determined by a combination of equipment-, matrix-, and solvent-dependent parameters. Key equipment-related factors include ultrasound power (acoustic power density), extraction time, sonotrode diameter and container configuration and size, and volume of the mixture. In addition, solvent type and affinity, and mixture factors such as solid-to-liquid ratio and by-product particles’ size also play a major role in the extraction efficiency [43,66]. Ultrasound energy can be delivered to the extraction mixture via water bath (indirect system) or by direct contact of a probe or sonotrode into the mixture [73].

Unlike previous reviews, the present study focuses primarily on processing by-products using ethanol-based food grade solvents. However, given the limited number of available studies, selected UAE studies from whole *Capsicum* fruits were also included. Table 2 shows results of studies of ultrasound extractions of *Capsicum* oleoresins (containing capsaicinoids) from chili pepper waste, and whole chili pepper fruit (first, second or third grade). Typically, ethanol-based solvent is mixed with the semi-solid chili pepper/chili pepper waste and the mixture is sonicated. Then, solvent is removed by evaporation, and a concentrated extract referred as *Capsicum* oleoresin is obtained. Among the studies reviewed, the oleoresin spiciness from by-product ranged between 6880 and 953,920 SHU, while 10,000 to 360,000 SHU oleoresins were obtained from whole pepper fruit extractions, depending on the ultrasound extraction conditions and chili pepper variety. This indicates similar or higher concentration of capsaicinoids in processing pepper by-products. Toscano et al. [43] optimized probe ultrasound-assisted extraction conditions for Red Habanero chili pepper waste (peels, placenta and seeds) and determined optimum extraction conditions associated with the highest capsaicinoid recovery (26% yield, 465,920 SHU) in the studied matrix (8 min 0.87 W/mL sonication with 96% ethanol, reaching 65 °C in the end of the process). The 8 min ultrasound-assisted extraction also demonstrated significant improvements in efficiency, achieving approximately 95% energy savings compared with the 5 h Soxhlet extraction [43]. The same optimized conditions were applied to the Carolina Reaper by-product (peels, placentas and seeds) and an extract/oleoresin (26% yield dry basis) with 59.62 mg/g (dry basis) was obtained, which corresponds to 953,920 SHU [48]. This magnitude of pungency is comparable to commercial oleoresins which have 500,000–1,500,000 SHU. Using the same ultrasound extraction conditions, temperature, solid to liquid ratio and solvent, an oleoresin with 348,800 SHU was registered for the Jalapeño chili pepper variety (28% yield) [48]. Another study employing indirect water bath sonication (100 W, 30 min, 50 °C) of Red Habanero dried slices of second- or third-grade chili pepper fruits mixed with 70% ethanol (1:5 *w*/*w*) resulted in 31% yield and approximately nine times less spicy oleoresin with 52,640 SHU [66]. However, comparison with the higher pungency values reported for Red Habanero processing by-products extracted by direct probe sonication [43] should be interpreted cautiously, given the differences in the extraction method and conditions (Table 2).

With respect to Jalapeño, non-optimized probe ultrasound extraction from peels, placentas and seeds by-product for 10 min with ethanol in 1:8 solid to liquid ratio resulted in 19% yield (dry basis) and 76,000 SHU [67], while optimized 14 mm probe ultrasound extraction conditions with the same type of by-product using 8 min, 65 °C final temperature, 150 W equivalent to power density of 0.87 W/mL (~175 mL mixture with 96% ethanol) resulted in a much higher yield (28% dry basis) and spiciness (348,800 SHU) [48]. Civan and Kumcuoglu [77] optimized the 14 mm probe ultrasound extraction conditions from dried and milled paste obtained from previously boiled Red Jalapeño peppers without seeds, using olive oil as an alternative solvent (20 g + 50 mL). Under optimum sonication conditions (160 W during 15 min at 40 °C, 2.3 W/mL power density) an oleoresin with approximately 17,600 SHU was obtained, a much lower value than the extract produced with faster sonications (8–10 min) from pepper by-product of same variety (Jalapeño) using ethanol solvent, similar power but a lower power density.

Overall, 96% ethanol seems to be a better option for extraction than pure ethanol. The use of moderate temperature (65 °C) as opposed to room temperature for ultrasound extraction also seems to increase the capsaicin concentration and corresponding spiciness in the final extract obtained after solvent evaporation. The sonication time was optimized by Toscano et al. (2025) in 8 min [43], but the time is very dependent on the ultrasound system used (bath vs. probe) as well as sonication temperature and ultrasound power density (W/mL) delivered to the mixture. Differences among cultivars, ultrasound method of extraction and ultrasound conditions prevent definitive conclusions regarding the influence of each parameter on oleoresin spiciness. Collectively, the available evidence indicates that UAE is capable of producing capsaicinoid-rich oleoresins with commercially relevant pungency levels while significantly reducing extraction time in comparison with conventional techniques. Several studies employing probe systems reported higher extraction efficiencies than ultrasonic bath configurations. Nevertheless, the substantial variability in extraction conditions among ultrasound studies highlights the need for greater harmonization of methodologies and reporting criteria, to facilitate comparison between studies and future industrial implementation [43,66,76].

## 4. Conclusions and Recommendations for Future Work

The growing demand for natural spicy ingredients and more sustainable food production systems creates new opportunities for the valorisation of chili pepper processing by-products. This review demonstrates that chili pepper residues, particularly those containing placental tissues, represent valuable sources of capsaicinoids that can be recovered and transformed into high-value *Capsicum* oleoresins, contributing to waste reduction and circular bioeconomy strategies within the spicy food sector.

Among the extraction technologies currently available, ultrasound-assisted extraction emerges as a particularly promising approach for the recovery of capsaicinoid-rich oleoresins from chili pepper by-products. The reviewed studies indicate that UAE can substantially reduce extraction times while producing oleoresins with commercially relevant pungency levels and extraction yields. Regarding the extractions from chili pepper processing by-products, oleoresin yields reaching 24 to 28% and pungency values reaching 953,920 SHU were reported, demonstrating the potential to obtain products that are comparable to commercial spicy ingredients. Food-grade ethanol-based extraction systems appear particularly attractive for foods applications owing to their effectiveness. Nevertheless, the currently available evidence remains limited as it is derived predominantly from laboratory-scale studies employing eight different chili cultivars, presenting different by-product spiciness compositions, two different ultrasound systems (bath vs. probe), different solid-to-liquid ratio (1:5 up to 1:50 dry basis), and a wide range of processing conditions including ultrasound power (100–480 W) and extraction temperature (35–65 °C). In addition, some studies did not provide detailed information about the UAE conditions applied. This methodological heterogeneity complicates cross-study comparisons and limits the establishment of robust processing recommendations. Consequently, UAE should be regarded as a promising technology rather than an established industrial solution.

Future research should focus on the standardization of extraction protocols, process scale-up (e.g., issues such as the attenuation of the cavitation field in larger vessels, probe erosion and metal contamination should also be investigated), solvent recovery, techno-economic evaluation and environmental assessment through life-cycle analysis. The development of standardized extraction protocols and quality indicators for *Capsicum* oleoresins would facilitate technology transfer and industrial adoption. Additional studies are also required to evaluate spicy *Capsicum* oleoresin ingredient functionality, sensory acceptance, storage stability, regulatory compliance and safety aspects in commercial food applications. The integration of UAE into extraction systems, together with stabilization strategies such as encapsulation, may further enhance the industrial applicability of capsaicinoid-rich oleoresins.

Overall, the available evidence supports the potential of UAE-based valorisation strategies to transform chili pepper processing waste from a disposal challenge into a sustainable source of food ingredients and other value-added products.

## Figures and Tables

**Table 1:** Biological properties of capsaicinoids and applications of spicy *Capsicum* oleoresin to food, agriculture and pharmaceutical industries.

Application Domain	Material Studied	Biological Properties	Reported Key Effect	Reference
**Food flavor enhancer**	Oleoresin	Spicy sensation	Enhance food sensory properties; production of spicy food products, condiments and sauces.	[26]
		Spiciness, pungent character, and color factor	The addition of oleoresin into avocado oil attributed both spicy character and red/orange color.	[27]
			Oleoresin flavor improved overall sensory characteristics of beef hamburger.	[28]
	Capsaicin	Salty taste substitute	Boosted salty taste cutting sodium by 5.67%, enabling low-Na food without losing flavor.	[29]
**Food preservative** **potential**	Oleoresin	Antimicrobial properties	Oleoresin displayed bacteriostatic effects on *E. coli* (1–3%, *w*/*w*) and on *L. innocua* (3% *w*/*w*), whereas pure capsaicin displayed no effects at the same tested concentrations.	[30]
		Antimicrobial properties on foodborne pathogens	Effective against *Staphylococcus aureus*, *Bacillus subtilis*, *Escherichia coli*, *Pseudomonas aeruginosa*, *Candida albicans*, *Aspergillus niger*; MIC values ranging from <1 to 100 µg/mL against foodborne pathogens.	[31]
		Microbial cell membrane disruption	The antimicrobial mechanism was elucidated, showing inhibition of peptidoglycan synthesis regulatory factors in *Staphylococcus aureus*, promoting microbial cell membrane disruption.	[32]
		Meat preservation against foodborne pathogens	Limited the growth of aerobic microorganisms (*S. aureus*, *Salmonella* spp., *B. thermosphacta*), molds and yeasts, lactic acid bacteria and coliforms.	[28]
**Food packaging** **material**	Capsaicin	Antimicrobial food packaging films	Endowed DAS/PVA film with excellent antimicrobial activity against both *S. aureus* and *E. coli*. The films were tested for packaging fresh-green grapes, prolonging their shelf life.	[33]
**Food antioxidant**	Oleoresin	Sunflower oil shelf-life enhancement	The addition of oleoresin (300 ppm) on sunflower oil extended the mixture’s shelf-life, exhibiting lower rancidity (−18%) in comparison to pure sunflower oil, after 8 h of heat.	[34]
		Antioxidant effect on meat fatty acids	Oleoresin applied on beef hamburgers delayed meat lipid oxidation in about 51.25% during a 15-day experiment when compared to the control (without the oleoresin).	[28]
**Agricultural crop** **protection**	Oleoresin	Pesticide potential	The average death time of American cockroaches (*Periplaneta americana*) using 50–100% concentration of oleoresin varied from 83.1 min to just 29.5 min.	[35]
	Capsaicin	Antifungal and insecticidal	Control pathogenic fungi growth in plant crops, as eco-friendly alternatives to pest management, and assist in the conservation and long-term storage of agrifood products.	[36,37]
**Human health: nutraceutical**	Capsaicin	Analgesic, anesthetic and anti-inflammatory	Protection against oxidative stress and inflammation; strong analgesic properties and anesthetic effects; scavenging of reactive oxygen species.	[38,39,40]
		Thermogenesis	Induction of metabolic acceleration and fat reduction following ingestion; anti-obesity effects.	[17,41,42]

**Table 2:** *Capsicum* oleoresin ultrasound-assisted extraction with ethanol-based solvent from by-products of chili pepper sauces processing and whole unprocessed chili peppers: effect of extraction conditions on oleoresin yield, capsaicinoids content and corresponding spiciness intensity ^a^.

Ultrasound System Type	Chili Pepper Variety/Species and Type of Waste	Sonication Time (min)	Sonication Temperature (°C) ^#^	Solid-to-Liquid Ratio (Ethanol Concentration)	Oleoresin Yield (%)	Capsaicin + Dihydrocapsaicin Concentration (mg/g)	Spiciness in Scoville Heat Units (SHU) *	Reference
**PROCESSING BY-PRODUCTS**								
Water bath (40 kHz, 5 g fresh waste + 50 mL solvent)	Mora (*Capsicum annuum*) *Pulp waste: pericarp, veins and stem*	20	35	1:10 (*w*/*v* wet basis) (96% ethanol)	~24 (wet basis)	1.87 (wet basis)	29,920	[74]
	Guajillo (*Capsicum annuum*) *Pulp waste: pericarp, veins and stem*	20	35	1:10 (*w*/*v* wet basis) (96% ethanol)	~16 (wet basis)	0.43 (wet basis)	6880	[74]
Probe (US conditions not reported)	Jalapeño (*Capsicum annuum*) *Peels, placentas and seeds*	10	nr	1:8 (*w*/*v*) (100% ethanol)	19 (dry basis)	4.74 (dry basis)	76,000	[67]
Probe (150 W, 26 kHz, 0.87 W/mL, 14 mm diameter probe, 25 g fresh waste + 150 mL solvent)	Carolina Reaper (*Capsicum chinense*) *Peels, placentas and seeds*	8	65 (final temperature	1:20 (*w*/*v* dry basis) (96% ethanol)	26 (dry basis)	59.62 (dry basis)	953,920	[48]
	Red Habanero (*Capsicum chinense*) *Peels, placentas and seeds*	8	65 (final temperature)	1:20 (*w*/*v* dry basis) (96% ethanol)	26 (dry basis)	29.12 (dry basis)	465,920	[43]
	Jalapeño (*Capsicum annuum*) *Peels, placentas and seeds*	8	65 (final temperature)	1:20 (*w*/*v* dry basis) (96% ethanol)	28 (dry basis)	21.80 (dry basis)	348,800	[48]
**WHOLE PEPPER (FIRST, SECOND OR THIRD GRADE FRUITS)**								
Water bath (480 W, 5 g of sample + 25 mL solvent)	Calabrian (*Capsicum annuum*) *Dried and finely grinded*	20	40	1:25 (*w*/*v* dry basis) (100% ethanol)	21 (dry basis)	8.12 (dry basis)	129,920	[75]
				1:25 (*w*/*v* dry basis) (70% ethanol)	26 (dry basis)	4.41 (dry basis)	70,560	[75]
Water bath (280 W, 5.6 W/mL, 1 g of sample + 50 mL of solvent)	Bode (*Capsicum chinense*) *Freeze-dried powder*	60	40 (final temperature)	1:50 (*w*/*v* dry basis) (70% ethanol)	nr	14.38 (dry basis)	230,000	[76]
	Malagueta (*Capsicum frutescens*) *Freeze-dried powder*	60	40 (final temperature)	1:50 (*w*/*v* dry basis) (70% ethanol)	nr	16.25 (dry basis)	260,000	[76]
	Habanero (*Capsicum chinense*) *Freeze-dried powder*	60	40 (final temperature)	1:50 (*w*/*v* dry basis) (70% ethanol)	nr	22.50 (dry basis)	360,000	[76]
Water bath (100 W, sample weight not reported)	Red Habanero (*Capsicum chinense*) *Dried slices of second or third grade fruits*	30	50	1:5 (*w*/*v* dry basis) (70% ethanol)	31 (dry basis)	3.29 (dry basis)	52,640	[66]
Probe (130 W, 12 mm probe diameter, 2 g + 20 mL of solvent)	Xkatik (*Capsicum annuum*) *Dried powder*	10	nr	1:10 (*w*/*v* dry basis) (70% ethanol)	nr	0.625 (dry basis)	10,000	[57]

^a^ nr means not reported; * Mean values of Scoville Heat Units (SHU) estimated from the equation: SHU = capsaicinoids (mg/g) × 16,000. ^#^ For Toscano et al. sonication was conducted with a sonotrode, with direct contact of the probe with the sonication mixture. At the start of sonication, the mixture was at room temperature and during the sonication the temperature increased, and the temperature shown is the final temperature at the end of sonication process [43,48].

## Data Availability

No new data were created or analyzed in this study. Data sharing is not applicable to this article.
